# Prediction of the histologic upgrade of ductal carcinoma *in situ* using a combined radiomics and machine learning approach based on breast dynamic contrast-enhanced magnetic resonance imaging

**DOI:** 10.3389/fonc.2022.1032809

**Published:** 2022-11-02

**Authors:** Hyo-jae Lee, Jae Hyeok Park, Anh-Tien Nguyen, Luu-Ngoc Do, Min Ho Park, Ji Shin Lee, Ilwoo Park, Hyo Soon Lim

**Affiliations:** ^1^ Department of Radiology, Chonnam National University Hospital, Gwangju, South Korea; ^2^ Department of Medicine, Chonnam National University, Gwangju, South Korea; ^3^ Department of Radiology, Chonnam National University, Gwangju, South Korea; ^4^ Department of Surgery, Chonnam National University Hwasun Hospital, Hwasun, South Korea; ^5^ Department of Pathology, Chonnam National University Hwasun Hospital, Hwasun, South Korea; ^6^ Department of Artificial Intelligence Convergence, Chonnam National University, Gwangju, South Korea; ^7^ Department of Data Science, Chonnam National University, Gwangju, South Korea; ^8^ Department of Radiology, Chonnam National University Hwasun Hospital, Hwasun, South Korea

**Keywords:** ductal carcinoma *in situ*, magnetic resonance imaging, machine learning, radiomics analysis, histologic upgrade, support vector machine

## Abstract

**Objective:**

To investigate whether support vector machine (SVM) trained with radiomics features based on breast magnetic resonance imaging (MRI) could predict the upgrade of ductal carcinoma *in situ* (DCIS) diagnosed by core needle biopsy (CNB) after surgical excision.

**Materials and methods:**

This retrospective study included a total of 349 lesions from 346 female patients (mean age, 54 years) diagnosed with DCIS by CNB between January 2011 and December 2017. Based on histological confirmation after surgery, the patients were divided into pure (n = 198, 56.7%) and upgraded DCIS (n = 151, 43.3%). The entire dataset was randomly split to training (80%) and test sets (20%). Radiomics features were extracted from the intratumor region-of-interest, which was semi-automatically drawn by two radiologists, based on the first subtraction images from dynamic contrast-enhanced T1-weighted MRI. A least absolute shrinkage and selection operator (LASSO) was used for feature selection. A 4-fold cross validation was applied to the training set to determine the combination of features used to train SVM for classification between pure and upgraded DCIS. Sensitivity, specificity, accuracy, and area under the receiver-operating characteristic curve (AUC) were calculated to evaluate the model performance using the hold-out test set.

**Results:**

The model trained with 9 features (Energy, Skewness, Surface Area to Volume ratio, Gray Level Non Uniformity, Kurtosis, Dependence Variance, Maximum 2D diameter Column, Sphericity, and Large Area Emphasis) demonstrated the highest 4-fold mean validation accuracy and AUC of 0.724 (95% CI, 0.619–0.829) and 0.742 (0.623–0.860), respectively. Sensitivity, specificity, accuracy, and AUC using the test set were 0.733 (0.575–0.892) and 0.7 (0.558–0.842), 0.714 (0.608–0.820) and 0.767 (0.651–0.882), respectively.

**Conclusion:**

Our study suggested that the combined radiomics and machine learning approach based on preoperative breast MRI may provide an assisting tool to predict the histologic upgrade of DCIS.

## Introduction

Ductal carcinoma *in situ* (DCIS) is a noninvasive neoplastic lesion of the breast which accounts for 20% of screen-detected breast cancers (1). DCIS is characterized by the proliferation of malignant epithelial cells that are confined within basement membrane (2); therefore, it allows less-invasive treatment options compared to invasive ductal carcinoma (IDC), which usually involves axillary interventions. While core-needle biopsy (CNB) is one of the gold standard tools for diagnosing breast lesions, the preoperative diagnosis of DCIS determined by CNB with a relatively small caliber (generally, 14-gauge) presents a potential sampling error and may lead to the upstaging of DCIS to invasive disease in surgically excised specimens. The rate of histologic upgrade of DCIS to IDC at surgical excision has been reported to be 6–59% (3–5).

Standard treatment for DCIS is similar to that for early-stage invasive breast cancer, which consists of conserving surgery, wide local excision followed by radiotherapy, mastectomy, or possibly hormonal therapy (6); however, it remains to be debatable whether these regimens are overtreatment especially in women with pure DCIS or without invasive component (7–9). According to National Comprehensive Cancer Network (NCCN) guideline, sentinel lymph node biopsy (SLNB) is not recommended for DCIS when breast-conserving surgery is planned because of the low incidence of axillary involvement in pure DCIS (1–2%) (6, 10). In contrast, SLNB or axillary lymph node dissection (ALND) is essential in the case of DCIS upgrading to IDC. Due to these differences in treatment strategy between pure and upgraded DCIS, predicting occult invasive component within newly diagnosed DCIS is of great clinical importance for providing minimally harmful and more effective patient management.

Various breast imaging modalities, including mammography and magnetic resonance imaging (MRI), have been utilized to evaluate the preoperative factors that are predictive of upgrading of DCIS to IDC (11–14). Most of the previous efforts, however, have applied the qualitative analysis of imaging features. More recently, a quantitative imaging analysis method, called radiomics, has emerged as a promising tool for extracting a large number of quantitative features from medical imaging data (15). While a few studies have tried to utilize the radiomics features for the prediction of upgrading of DCIS in limited efforts (16, 17), the extraction of large-scale radiomics features from imaging data and the training of a machine learning classifier using these features will add knowledge to the prior findings and help to evaluate the potential of this method for noninvasive classification between pure and upgraded DCIS.

The aim of our study was to investigate the feasibility of using a combined radiomics and machine learning approach for differentiating upgraded DCIS from pure DCIS based on breast MR images in patients diagnosed with DCIS by CNB.

## Materials and methods

### Study population and clinicopathologic features

**Figure 1:**
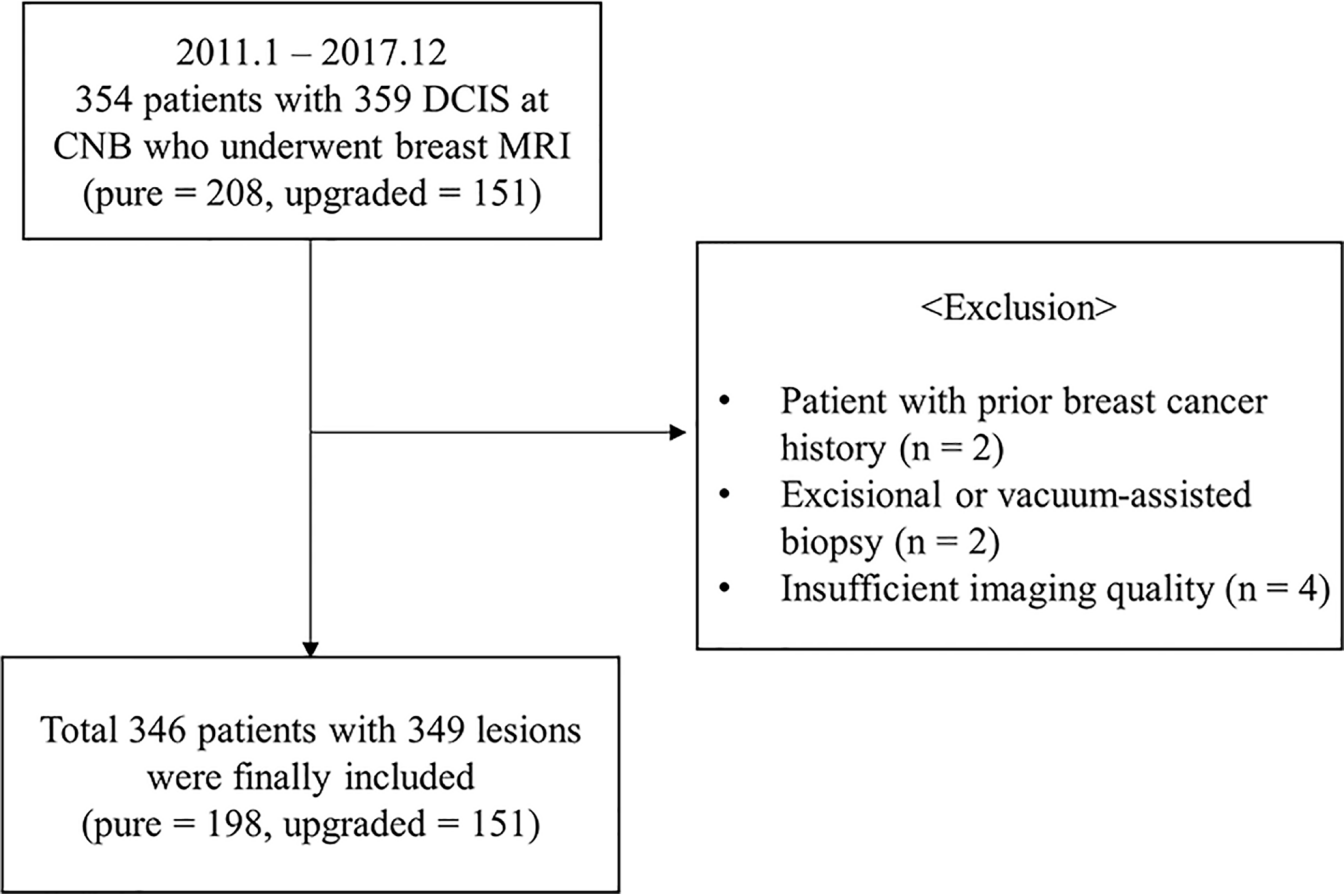
Flowchart of study population with exclusion criteria.

This retrospective study was approved by the institutional review board of our center (IRB No. 2022-110), and the informed consent was waived. We selected a patient cohort with the diagnosis of CNB-proven DCIS from the pathologic reports between January 2011 and December 2017. 354 female patients who underwent a preoperative breast MRI and the surgical resection were eligible for this study. The following criteria were applied for the exclusion of patients: 1) prior surgery or radiation therapy for breast cancer (n = 2), 2) prior excision or vacuum-assisted biopsy (n = 2), 3) preoperative breast MRI with an insufficient imaging quality or a poor delineation of tumor due to prominent background parenchymal enhancement (n = 4). After these exclusions, a total of 346 women (mean age ± standard deviation [SD], 54 ± 10.5 years-old; range, 22−81 years-old) with 349 lesions (upgraded DCIS, n = 151; pure DCIS, n = 198) were included (**Figure 1**).

Clinicopathologic factors, including patients’ age and symptom at diagnosis, mammography (mass, microcalcifications only, asymmetry, occult), ultrasound (mass, non-mass), and MR morphology (mass, non-mass enhancement), the size of enhancing portion at MRI, the type of surgery, pathologic diagnosis (upgraded DCIS or pure DCIS), Ki-67 proliferation rate, the pathologic size of DCIS and invasive cancer, axillary lymph node metastasis, and molecular subtype by immunohistochemical staining, were reviewed. The lesions that were difficult to be classified as “mass” on ultrasound but identified as an area of altered echotexture were denoted as “non-mass”. CNB was performed with a 14-gauge biopsy gun and at least 4 samples were harvested from each patient. All pathologic information after surgery were obtained from the pathology reports of surgical specimen. Based on the histopathologic analysis of surgical specimen, the patients were categorized into either pure DCIS or upgraded DCIS. The upgraded DCIS was histologically defined as having microinvasive or invasive foci within a tumor.

### MRI acquisition

All patients underwent bilateral breast MRI using three different 3-tesla scanners (Tim trio, Skyra, or Skyra II; Siemens Healthcare, Erlangen, Germany) with a dedicated 16- or 18-channel breast coil. Dynamic contrast-enhanced (DCE)-MRI was performed using T1-weighted axial 3-dimensional fat-saturated spoiled gradient-echo (TR/TE = 4.5/1.7 ms, matrix size = 448×358 mm, FOV = 320×320 mm^2^, and slice thickness = 1.5 mm) with the administration of gadoterate meglumine (Dotarem; Guerbet, Aulnay-sous-bois, France) at a dose of 0.1 mmol/kg body weight. DCE-MRI consisted of a pre-contrast and five post-contrast series. Subtraction images were generated by subtracting pre-contrast series from each of the five post-contrast series.

### Tumor segmentation

**Figure 2:**
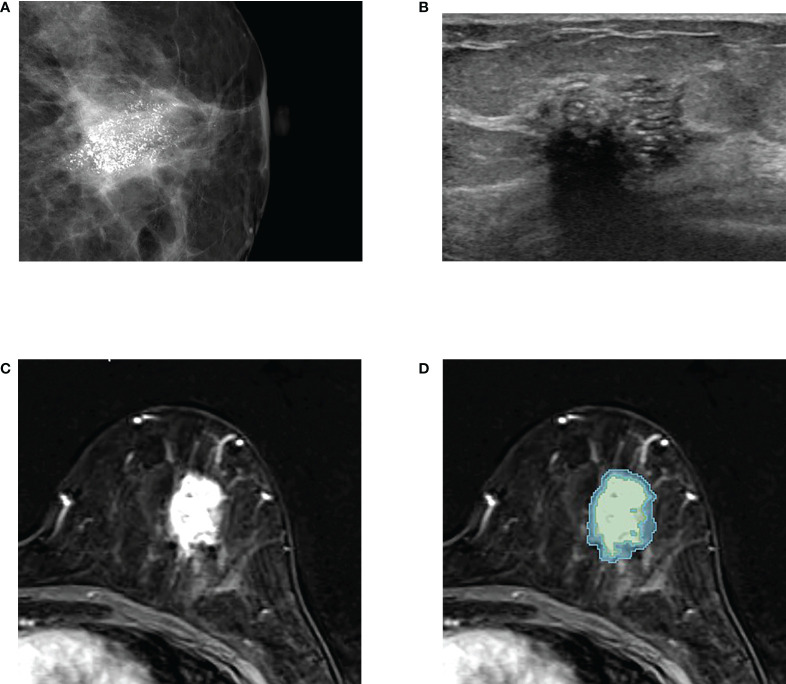
An example of tumor segmentation in a 50-year-old woman who complained of palpable lump in her left breast, which was pathologically confirmed as upgraded ductal carcinoma *in situ* (DCIS) after surgical excision. **(A)** Mammography shows about 3 cm extent of fine pleomorphic microcalcifications seen in the left central breast. **(B)** Ultrasound image shows the non-mass lesion with microcalcifications. **(C)** Axial first postcontrast T1-weighted image with subtraction shows a 3 cm irregular heterogeneously enhancing mass in left lower central breast. **(D)** The green and blue shadings represent the intratumor and peritumor ROIs, respectively, which are created by 3D slicer.

**Figure 3:**
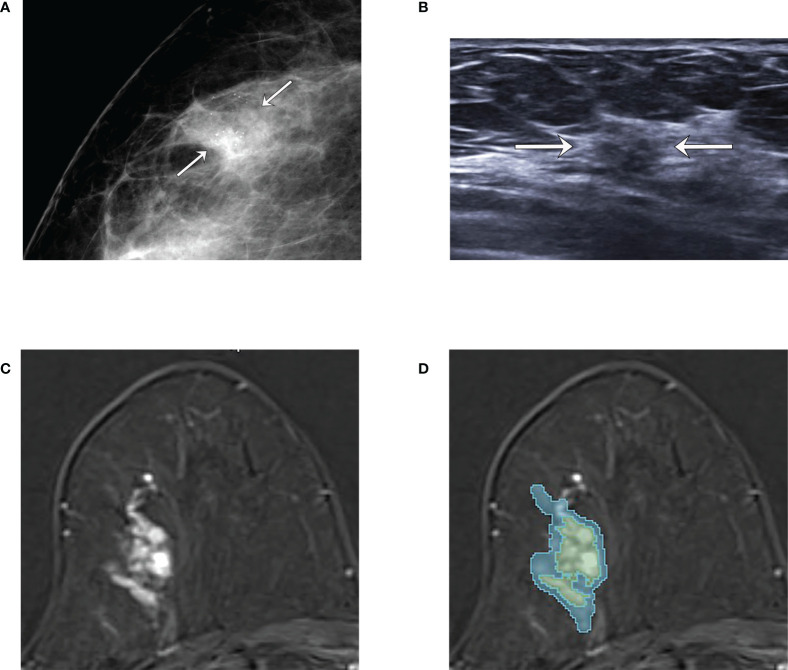
An example of tumor segmentation in a 66-year-old woman who complained of incidentally detected microcalcifications in the screening mammogram, which was pathologically confirmed as pure ductal carcinoma *in situ* (DCIS) after surgical excision. **(A)** Mammography shows focal asymmetry with grouped microcalcifications seen in the right outer breast (arrows). **(B)** Ultrasound image shows the subtle non-mass lesion with microcalcifications (arrows). **(C)** Axial first postcontrast T1-weighted image with subtraction shows about 3.1 cm extent of clumped non-mass enhancement in right upper outer breast. **(D)** The green and blue shadings represent the intratumor and peritumor ROIs, respectively, which are created by 3D slicer.

The first subtraction images were utilized for the semi-automatic segmentation of tumor. Two radiologists (4 and 18 years of experience in breast imaging, respectively), who were blinded to the clinicopathologic data, reviewed the images and semi-automatically drew the intratumor region-of-interest (ROI) using an open-source software, 3D Slicer (https://www.slicer.org). A 5-mm peritumor ROI was automatically created by extending the boundary of the intratumor ROI using a built-in segmentation tool in 3D Slicer, and the combined ROI was obtained by merging intratumor- and donut-like peritumor-ROIs. The ROIs drawn on multiple slices were rendered into a 3D volume. The examples of tumor segmentation on the first subtraction T1-weighted image are shown in **Figures 2** and **3**.

### Data preprocessing and radiomics feature extraction

In order to account for the differences in pixel intensities across different MR scanners, the pixel intensity values on the subtraction images were normalized using Z-score (18). Each pixel value was normalized using the following equation:


(1) $$z_{i}=\frac{x_{i}-m_{R}}{\sigma_{R}}$$

, where *z*_*i*_ , *x*_*i*_ , *m*_*R*_ , and *σ*_*R*_ stand for the normalized pixel value, original pixel value, and the mean and standard deviation of pixel values derived from the combined ROIs.

Following the pixel normalization, a total of 107 radiomics features were extracted from the intratumor ROIs using a Pyradiomics module in python package (19). The 107 features consist of three statistical properties: 14 shape, 18 intensity, and 75 texture features (**Supplemental Table**).

### Feature selection and classifier training

The entire dataset was randomly split into training (80%) and test sets (20%), with each cohort containing the approximately similar ratio of upgraded to pure DCIS. The training set was used for selecting features and training a classifier, while the hold-out test set was used for testing the performance of the classifier model.

In order to minimize a model complexity and choose an optimal set of features for classifier, a least absolute shrinkage and selection operator (LASSO) was applied. LASSO was based on L1 regularization and the coefficients of unimportant regression variables were rendered to be zero (20). Features with a bigger absolute coefficient value can be interpreted as having a greater relevance for classification. The three-quarter of the training set was randomly selected to fit LASSO. This process was repeated 500 times and the absolute coefficient values of the selected features were summed. Finally, the top 14 features with the highest accumulated sum of absolute coefficient values were selected as candidate features. The feature selection process was implemented using a python package, scikit-learn (21).

Based on the 14 candidate features that were selected, a support vector machine (SVM) algorithm with a Radial Basis Function kernel was utilized to build a classifier model using a scikit-learn package. In order to find the optimal set of features for the final classifier, the SVM models with every combination of the 14 selected features were tested using the training set with a 4-fold cross-validation method. Hyperparameters were optimized for each fold. The final classifier model was chosen based on the highest mean validation accuracy, area under the receiver operating characteristics curve (AUC) and the minimum standard deviation of the 4-fold model accuracies. In addition, the final classifier model must include the top-4 features based on the sum of accumulated LASSO coefficients. The performance of the final classifier model for distinguishing between the pure and upgraded DCIS was assessed using the hold-out test set.

### Statistical analysis

Interobserver reproducibility of the extracted features from the ROIs drawn by the two radiologists was analyzed by calculating an intraclass correlation coefficient (ICC) and categorized as excellent (>0.90), good (0.75–0.90), moderate (0.50–0.75), or poor (<0.50) (22). Clinicopathologic features were compared between the training and test sets using the independent t-test for the continuous variables and the Fisher’s exact test or Chi-square test for the categorical variables. Sensitivity (upgraded DCIS considered as a positive condition), specificity, accuracy, and AUC were calculated for assessing model performance.

## Results

### Patient characteristics and clinicopathologic features

The mean time interval between CNB and surgical excision was 25 days (range, 10–71 days). The following surgeries were performed: breast conserving surgery (n = 251, 71.9%) or mastectomy (n = 98, 28.1%). One hundred fifty-one out of 349 lesions that were included in this study (43.3%) were histologically determined to be upgraded to IDC, which was within the range of the previously reported rate of upgrading (23, 24).

The baseline characteristics and clinicopathologic features between the training (n = 279) and test sets (n = 70) are summarized in **Table 1**. There were no significant differences in baseline characteristics and clinicopathologic features between the two groups (all, p > 0.05).

**Table 1:** Comparison of baseline characteristics between training (n = 279) and test (n = 70) sets.

	Training	Test	P
Patient age (mean ± SD, years)	52.2 ± 10.4	50.9 ± 9.5	0.342
Symptom			0.089
Palpable lump	101 (36.2%)	20 (28.6%)	
Nipple discharge	23 (8.2%)	2 (2.9%)	
None	155 (55.6%)	48 (68.6%)	
Mammogram morphology			0.997
Mass	26 (9.3%)	7 (21.2%)	
Microcalcifications only	142 (50.9%)	35 (19.8%)	
Asymmetry	61 (21.9%)	15 (19.7%)	
Occult	50 (17.9%)	13 (20.6%)	
Ultrasound morphology			0.291
Mass	63 (22.6%)	12 (17.1%)	
Non-mass lesion	189 (67.7%)	54 (77.1%)	
No delineation	27 (9.7%)	4 (5.7%)	
MRI morphology			0.666
Mass	90 (32.3%)	20 (28.6%)	
Non-mass enhancement	189 (67.7%)	50 (71.4%)	
MR enhancing portion size (mean ± SD, mm)	37.3 ± 23.2	36.2 ± 21.2	0.692
Surgery type			0.183
Breast conserving surgery	196 (70.3%)	55 (78.6%)	
Mastectomy	83 (29.7%)	15 (21.4%)	
Pathologic diagnosis			0.938
Upgraded DCIS	121 (43.4%)	30 (42.9%)	
Pure DCIS	158 (56.6%)	40 (57.1%)	
Ki-67 (≥14%)			0.789
Yes	131 (47.0%)	31 (44.3%)	
No	148 (53.0%)	39 (55.7%)	
Pathologic size of DCIS (mean ± SD, mm)	28.1 ± 20.7	26.8 ± 15.9	0.627
Pathologic size of invasive component(mean ± SD, mm)	8.4 ± 11.1	6.4 ± 7.1	0.221
Axillary lymph node metastasis			0.820
Yes	16 (5.7%)	3 (4.3%)	
No	263 (94.3%)	67 (95.7%)	
IHC subtype			0.915
Luminal A	115 (41.2%)	31 (44.3%)	
Luminal B	59 (21.1%)	15 (21.4%)	
HER2-positive	83 (29.7%)	18 (25.7%)	
TNBC	22 (8.0%)	6 (8.6%)	

MRI, magnetic resonance imaging; DCIS, ductal carcinoma *in situ*; IHC, immunohistochemistry; HER2, human epidermal growth factor receptor 2; TNBC, triple-negative breast cancer; SD, standard deviation.

The comparison of baseline characteristics and clinicopathologic features between the pure and upgraded DCIS is shown in **Table 2**. The patients in the upgraded DCIS group were significantly younger (p = 0.021; mean age in the pure and upgraded DCIS = 53.0 and 50.5 years-old, respectively), had more symptoms of palpable lump (p<0.001), higher Ki-67 proliferation rate (p = 0.001), and more triple-negative breast cancer (TNBC) and less luminal A subtype (p = 0.042) than pure DCIS (**Figures 2** and **3**). No patients in the pure DCIS group had axillary lymph node metastasis (p< 0.001).

**Table 2:** Comparison of clinicopathologic features between upgraded (n = 151) and pure DCIS (n = 198).

	Upgraded	Pure	P
Patient age (mean ± SD, years)	50.5 ± 9.3	53.0 ± 10.8	0.021
Symptom			< 0.001
Palpable lump	70 (46.4%)	51 (26.0%)	
Nipple discharge	12 (7.9%)	13 (6.6%)	
None	69 (45.7%)	132 (67.3%)	
Mammogram morphology			0.120
Mass	20 (13.2%)	13 (6.6%)	
Microcalcifications only	76 (50.3%)	101 (51.0%)	
Asymmetry	33 (21.9%)	43 (21.7%)	
Occult	22 (14.6%)	41 (20.7%)	
Ultrasound morphology			0.093
Mass	33 (21.9%)	42 (21.2%)	
Non-mass lesion	99 (65.6%)	144 (72.7%)	
No delineation	19 (12.6%)	12 (6.1%)	
MRI morphology			0.563
Mass	45 (29.8%)	65 (32.8%)	
Non-mass enhancement	106 (70.2%)	133 (67.2%)	
MR enhancing portion size (mean ± SD, mm)	39.6 ± 20.4	35.2 ± 24.3	0.068
Surgery type			0.188
Breast conserving surgery	103 (68.2%)	148 (74.7%)	
Mastectomy	48 (31.8%)	50 (25.3%)	
Ki-67 (≥14%)			0.001
Yes	86 (57.0%)	76 (38.4%)	
No	65 (43.0%)	122 (61.6%)	
Pathologic size of DCIS (mean ± SD, mm)	35.0 ± 17.7	26.2 ± 19.9	0.003
Pathologic size of invasive component(mean ± SD, mm)	8.0 ± 10.5	N/A	
Axillary lymph node metastasis			< 0.001
Yes	19 (12.6%)	0 (0)	
No	132 (87.4%)	198 (100%)	
IHC subtype			0.042
Luminal A	58 (38.4%)	88 (44.4%)	
Luminal B	41 (27.2%)	33 (16.7%)	
HER2-positive	37 (24.5%)	64 (32.3%)	
TNBC	15 (9.9%)	13 (6.6%)	

MRI, magnetic resonance imaging; DCIS, ductal carcinoma *in situ*; IHC, immunohistochemistry; HER2, human epidermal growth factor receptor 2; TNBC, triple-negative breast cancer; SD, standard deviation; NA, Not Applicable.

### Interobserver reproducibility

The ICCs (mean ± SD) for the selected features were 0.937 ± 0.072 (range, 0.730–0.993) for intratumor ROIs and 0.9 ± 0.094 (range, 0.518–0.996) for peritumor ROIs, respectively, showing an excellent agreement between the two radiologists. The features based on the ROIs drawn by the first radiologist were used to train the model.

### Feature selection

**Figure 4:**
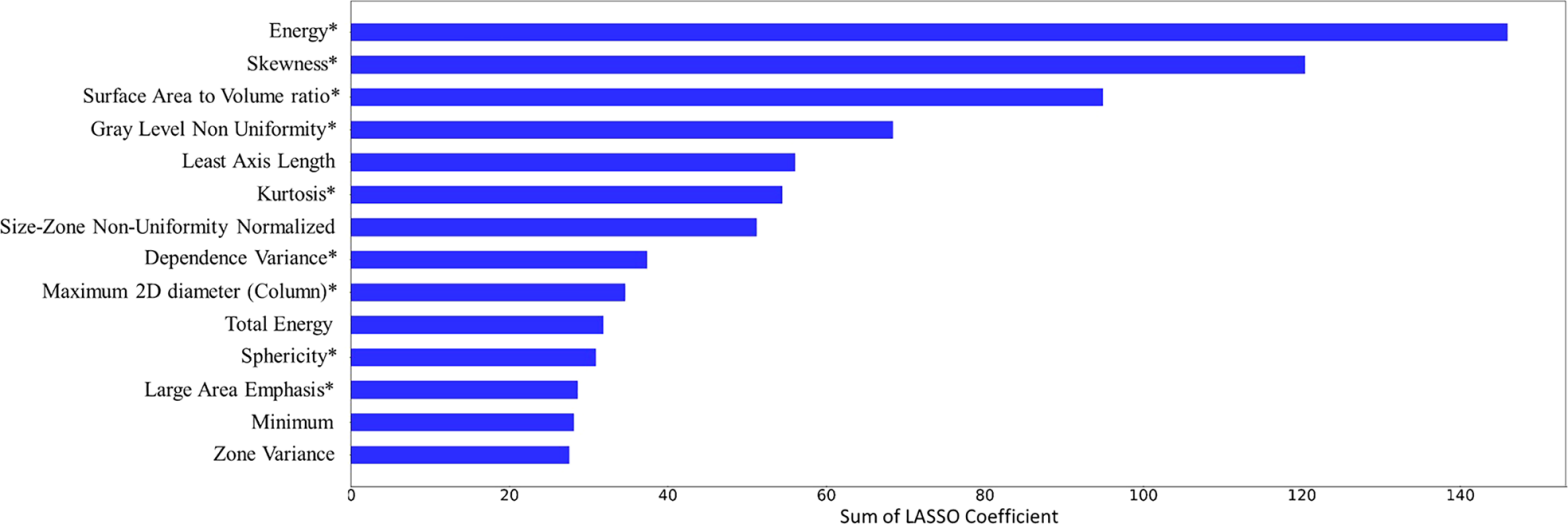
Feature selection results. 14 features and their accumulated sum of the least absolute shrinkage and selection operator (LASSO) coefficients over 500 iterations are plotted. 9 features that were included in the final model were marked with an asterisk (*).

The top 14 features with the highest accumulated sum of LASSO coefficients are shown in **Figure 4**. The 14 features consisted of 4 shape features (*Least Axis Length, Maximum 2D Diameter Column, Sphericity, Surface Volume Ratio*), 5 intensity features (*Energy, Kurtosis, Minimum, Total Energy, Skewness*), and 5 texture features (*Dependence Variance, Gray Level Non Uniformity, Large Area Emphasis, Size Zone Non Uniformity Normalized, Zone Variance*). The sums of accumulated LASSO coefficients for the top-4 features (*Energy, Skewness, Surface Area to Volume ratio, Gray Level Non Uniformity*) were relatively large compared to those of other features. Therefore, it was ensured that the final model included these four features.

### Model Performance

**Figure 5:**
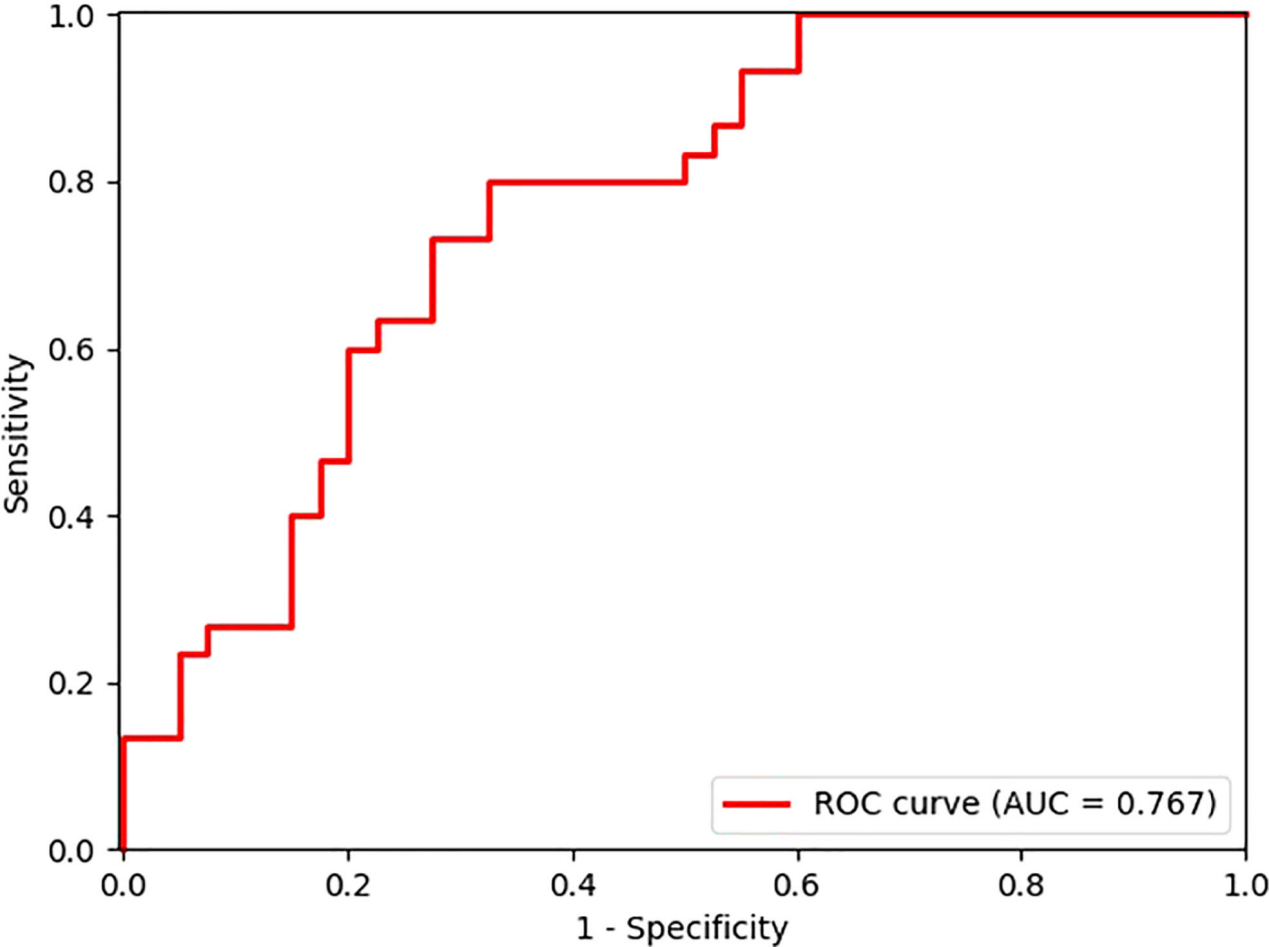
Receiver operating characteristic (ROC) curve of the model performance using the hold-out test set.

After exploring all potential models with the combination of the 14 candidate features, the model trained with 9 features, including *Energy, Skewness, Surface Area to Volume ratio, Gray Level Non Uniformity, Kurtosis, Dependence Variance, Maximum 2D diameter Column, Sphericity, and Large Area Emphasis* was chosen as the final classifier (**Figure 4**). This model demonstrated the highest 4-fold mean validation accuracy and AUC of 0.724 (95% CI, 0.619–0.829) and 0.742 (0.623–0.860), respectively. The accuracy and AUC of the final classifier using the hold-out test set were 0.714 (0.608–0.820) and 0.767 (0.651–0.882), respectively (**Table 3** and **Figure 5**). The sensitivity and specificity using the test set were 0.733 (0.575–0.892) and 0.7 (0.558–0.842), respectively. **Table 4** shows a confusion matrix that summarizes the performance of the final classifier.

**Table 3:** The summary of the radiomics model performance.

Validation set (average from 4-fold CV)				Test set			
Sensitivity	Specificity	AUC	Accuracy	Sensitivity	Specificity	AUC	Accuracy
0.587 (0.412–0.761)	0.829 (0.714–0.945)	0.742 (0.623–0.860)	0.724 (0.619–0.829)	0.733 (0.575–0.892)	0.7 (0.558–0.842)	0.767 (0.651–0.882)	0.714 (0.608–0.820)

Data are percentages, with 95% confidence intervals in parentheses.

AUC, area under the receiver operating characteristic curve; CV, cross validation; RA, radiomics analysis.

**Table 4:** Confusion matrix of the radiomics model in the test set.

		Pathologic outcome		Total
		Upgraded DCIS	Pure DCIS	
Observed outcome	Upgraded DCIS	22	12	34
Pure DCIS	8	28	36
Total		30	40	70

DCIS, ductal carcinoma *in situ*.

## Discussion

This study suggested that the combined radiomics and machine learning approach in breast MRI may provide a tool for the preoperative prediction of DCIS that has an increased risk of carrying an invasive component. Our proposed model demonstrated an accuracy of 0.714 and AUC of 0.767 for the prediction of DCIS upgraded to IDC.

The NCCN guideline does not recommend a routine SLNB for DCIS with a planned breast-conserving surgery, nor ALND in the absence of invasive cancer or proven axillary disease to minimize the chances of overtreating pure DCIS (6). Although DCIS has a rare axillary involvement, axillary evaluation may be considered in some DCIS lesions which are found to form a mass by clinical examination or imaging. Unfortunately, CNB can underestimate the occult invasive component in a relatively small-caliber specimen; therefore, many institutions have been utilizing SLNB for patients with DCIS, who undergo breast-conserving surgery, and many studies have attempted to noninvasively identify DCIS with occult invasive component or predict the upgrading of DCIS after surgical excision (23, 25–27).

MRI is the most sensitive tool to detect malignancy among various breast imaging tools (28). Several studies focused on finding meaningful discriminators or developing quantitative features from breast MRI for predicting the upgrade of DCIS (12, 16, 29–31). Park et al. reported that low T2 signal, heterogeneous or rim enhancement, and low ADC value in multiparametric breast MR may be helpful in predicting an upgrade of DCIS to IDC (12). In a multi-institutional retrospective study, palpable lump, BI-RADS category 4 or 5 on mammogram, mass formation on ultrasound, and tumor size more than 20 mm on MRI were independent predictors of upgrading of DCIS to IDC after surgery (32). Lee at al. utilized diffusion-weighted breast MRI in prediction of upstaging in women with biopsy-proven DCIS (33). Most of these previous studies, however, relied upon the conventional ways to analyze medical imaging data, which are based on the qualitative assessment of imaging parameters. A few reports have investigated the use of MRI-extracted quantitative features or applied deep learning algorithms for the prediction of the upgrading of DCIS (16, 34). Most recently, Mori et al. reported that radiomics features extracted from ultrafast MRI could effectively distinguish low-grade DCIS (n = 16) from non-low-grade DCIS (n = 37) or from upgraded DCIS (n = 33) (35). While these information obtained in an early contrast flow from ultrafast MRI can provide an useful information, ultrafast MRI may be insufficient for the morphological evaluation because of its relatively large voxel size compared to the conventional DCE-MRI (35). In addition, the conventional DCE-MRI is preferentially used for differentiating cancerous from benign tissue because it provides information regarding the change in blood flow with the broad range of time frame. In another study, Harowicz et al. analyzed 29 algorithmically assessed imaging features, including morphologic and texture features as well as qualitatively assessed MRI enhancement features from DCE-MRI from 131 patients for predicting the upgrade of DCIS (16). In comparison, our study extracted a large number of quantitative radiomics features, totaling 107, which were derived from the semi-automatically drawn ROIs of enhancing portion of the conventional DCE-MRI. Our study also represents a relatively large number of patient dataset (n = 346). In contrast to other studies where the proportion between pure and upgraded DCIS was imbalanced, we ensured to have relatively balanced dataset. The ratio of pure DCIS to upgraded DCIS in our study was 1.3 while it ranged from 2.1 to 2.7 in other studies (16, 35, 36).

In our study cohort, the rate of upgrade to invasive cancer was 43.3%, which was within the range of the previously reported rates (6–59%) (4, 5). In a previous meta-analysis, the high underestimation of DCIS at CNB was associated with the use of 14-gauge needle (versus 11-gauge vacuum-assisted device), high histologic grade lesion (versus non-high grade lesion), lesion size more than 20 mm at imaging, BI-RADS score of 4 or 5, mass at mammography (versus microcalcification only), and palpability (23). Our study included relatively large lesions in both groups (mean size, 39.6 mm and 35.2 mm for upgraded and pure DCIS, respectively). In addition, most of the biopsies were performed using 14-gauge device and patients who underwent vacuum-assisted biopsy were excluded. We speculate that these factors may have influenced the performance of our model.

There are several limitations in this study. Although we utilize the semi-automatic segmentation tool from the open-source software, this process is time-consuming and needs significant human efforts. In addition, interobserver reproducibility is one of the most important aspects in the extraction of radiomics features from ROI. Although our results showed an excellent interobserver reproducibility from the two radiologists, the development of automatic ROI segmentation method will enhance the efficiency and reproducibility in future efforts. Second, it was a retrospective study without an external validation test. Although we have performed the feature selection with the random split of training set 500 times and allocated the hold-out test set to evaluate the performance of our model, future studies with an external validation incorporating multicenter patient data will prove the validity of our method. The collection of multicenter patient data from our collaborating hospitals are currently underway. Third, we used only subtraction images to define tumor and extract radiomics features. Because DCIS lesions are usually revealed as non-mass enhancement in MR, it is difficult to delineate tumor boundary from background parenchymal tissue in T2-weighted images. In addition, the radiomics features from the intratumor ROI were only utilized for classification, and the combined ROI (intratumor + peritumor) was used only for the purpose of pixel intensity normalization. As the peritumoral region beyond contrast enhancement is believed to contain critical information regarding tumor microenvironment (37), further research is required to properly define and delineate the peritumoral region, especially, for non-mass enhancement lesions, and to assess its effect on the predictive performance of radiomics model using multi-parametric MRI data.

In conclusion, our study demonstrated that the combined radiomics and machine learning approach based on preoperative breast MRI may provide an assisting tool to predict the histologic upgrade of DCIS after surgical excision. The proposed method can help clinicians to provide an optimal medical care to patients with underestimated invasive breast cancer.

## Data availability statement

The original contributions presented in the study are included in the article/**Supplementary Material**. Further inquiries can be directed to the corresponding authors.

## Ethics statement

The studies involving human participants were reviewed and approved by the Institutional review board of Chonnam National University Hospital. Written informed consent for participation was not required for this study in accordance with the national legislation and the institutional requirements.

## Author contributions

H-JL and HL: study design. H-JL, HL, and IP: study conduct. JP, MP, JL: data collection and clinical data support. JP, L-ND, A-TN, and IP: data processing and interpretation using machine learning. JP, L-ND, A-TN, and IP: statistical analysis. H-JL and HL: MRI reading. H-JL and JP: drafting manuscript. All authors contributed to the article and approved the submitted version.

## Funding

This study was supported by grants from the Ministry of Education, Republic of Korea (NRF-2022R1I1A3072856) and Chonnam National University Hospital Biomedical Research Institute (BCRI22037 & BCRI22081).

## Conflict of interest

The authors declare that the research was conducted in the absence of any commercial or financial relationships that could be construed as a potential conflict of interest.

## Publisher’s note

All claims expressed in this article are solely those of the authors and do not necessarily represent those of their affiliated organizations, or those of the publisher, the editors and the reviewers. Any product that may be evaluated in this article, or claim that may be made by its manufacturer, is not guaranteed or endorsed by the publisher.

## References

[1] Ernster VL Ballard-Barbash R Barlow WE Zheng Y Weaver DL Cutter G . Detection of ductal carcinoma *in situ* in women undergoing screening mammography. J Natl Cancer Institute. (2002) 94(20):1546–54. doi: 10.1093/jnci/94.20.1546 12381707

[2] Virnig BA Tuttle TM Shamliyan T Kane RL . Ductal carcinoma *in situ* of the breast: a systematic review of incidence, treatment, and outcomes. J Natl Cancer Institute. (2010) 102(3):170–8. doi: 10.1093/jnci/djp482 20071685

[3] Page DL Dupont WD Rogers LW Jensen RA Schuyler PA . Continued local recurrence of carcinoma 15–25 years after a diagnosis of low grade ductal carcinoma *in situ* of the breast treated only by biopsy. Cancer (1995) 76(7):1197–200. doi: 10.1002/1097-0142(19951001)76:7<1197::AID-CNCR2820760715>3.0.CO;2-0 8630897

[4] Knuttel F Menezes G Van Diest P Witkamp A Van Den Bosch M Verkooijen H . Meta-analysis of the concordance of histological grade of breast cancer between core needle biopsy and surgical excision specimen. J Br Surg (2016) 103(6):644–55. doi: 10.1002/bjs.10128 26990850

[5] Dillon MF McDermott EW Quinn CM O'Doherty A O'Higgins N Hill AD . Predictors of invasive disease in breast cancer when core biopsy demonstrates DCIS only. J Surg Oncol (2006) 93(7):559–63. doi: 10.1002/jso.20445 16705731

[6] Gradishar WJ Anderson BO Balassanian R Blair SL Burstein HJ Cyr A . Breast cancer version 2.2015. J Natl Compr Cancer Network J Natl Compr Canc Netw (2015) 13(4):448–75. doi: 10.6004/jnccn.2015.0060 25870381

[7] Elshof LE Tryfonidis K Slaets L van Leeuwen-Stok AE Skinner VP Dif N . Feasibility of a prospective, randomised, open-label, international multicentre, phase III, non-inferiority trial to assess the safety of active surveillance for low risk ductal carcinoma *in situ*–the LORD study. Eur J Cancer (2015) 51(12):1497–510. doi: 10.1016/j.ejca.2015.05.008 26025767

[8] Hwang ES Hyslop T Lynch T Frank E Pinto D Basila D . (Comparison of operative versus monitoring and endocrine therapy) trial: A phase III randomised controlled clinical trial for low-risk ductal carcinoma *in situ* (DCIS). BMJ Open (2019) 9(3):e026797. doi: 10.1136/bmjopen-2018-026797 PMC6429899 30862637

[9] Francis A Thomas J Fallowfield L Wallis M Bartlett JM Brookes C . Addressing overtreatment of screen detected DCIS; the LORIS trial. Eur J cancer. (2015) 51(16):2296–303. doi: 10.1016/j.ejca.2015.07.017 26296293

[10] McMasters KM Chao C Wong SL Martin RC III Edwards MJ . Sentinel lymph node biopsy in patients with ductal carcinoma *in situ*: A proposal. Cancer (2002) 95(1):15–20. doi: 10.1002/cncr.10641 12115311

[11] Kim G Mikhael PG Oseni TO Bahl M . Ductal carcinoma *in situ* on digital mammography versus digital breast tomosynthesis: rates and predictors of pathologic upgrade. Eur Radiol (2020) 30(11):6089–98. doi: 10.1007/s00330-020-07021-2 PMC7572611 32591884

[12] Park AY Gweon HM Son EJ Yoo M Kim JA Youk JH . Ductal carcinoma *in situ* diagnosed at US-guided 14-gauge core-needle biopsy for breast mass: preoperative predictors of invasive breast cancer. Eur J Radiol (2014) 83(4):654–9. doi: 10.1016/j.ejrad.2014.01.010 24534119

[13] Huang Y-T Cheung Y-C Lo Y-F Ueng S-H Kuo W-L Chen S-C . MRI Findings of cancers preoperatively diagnosed as pure DCIS at core needle biopsy. Acta Radiol (2011) 52(10):1064–8. doi: 10.1258/ar.2011.110213 21969708

[14] Goto M Yuen S Akazawa K Nishida K Konishi E Kajihara M . The role of breast MR imaging in pre-operative determination of invasive disease for ductal carcinoma *in situ* diagnosed by needle biopsy. Eur Radiol (2012) 22(6):1255–64. doi: 10.1007/s00330-011-2357-2 22205445

[15] Gillies RJ Kinahan PE Hricak H . Radiomics: Images are more than pictures, they are data. Radiology (2016) 278(2):563–77. doi: 10.1148/radiol.2015151169 PMC4734157 26579733

[16] Harowicz MR Saha A Grimm LJ Marcom PK Marks JR Hwang ES . Can algorithmically assessed MRI features predict which patients with a preoperative diagnosis of ductal carcinoma *in situ* are upstaged to invasive breast cancer? J Magn Reson Imaging (2017) 46(5):1332–40. doi: 10.1002/jmri.25655 PMC5910028 28181348

[17] Shi B Grimm LJ Mazurowski MA Baker JA Marks JR King LM . Can occult invasive disease in ductal carcinoma *In situ* be predicted using computer-extracted mammographic features? Acad Radiol (2017) 24(9):1139–47. doi: 10.1016/j.acra.2017.03.013 PMC5557686 28506510

[18] Haga A Takahashi W Aoki S Nawa K Yamashita H Abe O . Standardization of imaging features for radiomics analysis. J Med Invest (2019) 66(1.2):35–7. doi: 10.2152/jmi.66.35 31064950

[19] van Griethuysen JJM Fedorov A Parmar C Hosny A Aucoin N Narayan V . Computational radiomics system to decode the radiographic phenotype. Cancer Res (2017) 77(21):e104–e7. doi: 10.1158/0008-5472.CAN-17-0339 PMC5672828 29092951

[20] Ranstam J Cook J . LASSO regression. J Br Surg (2018) 105(10):1348. doi: 10.1002/bjs.10895

[21] Pedregosa F Varoquaux G Gramfort A Michel V Thirion B Grisel O . Scikit-learn: Machine learning in Python. J Mach Learn Res (2011) 12:2825–30. doi: 10.48550/ARXIV.1201.0490

[22] Bartko JJ . The intraclass correlation coefficient as a measure of reliability. psychol Rep (1966) 19(1):3–11. doi: 10.2466/pr0.1966.19.1.3 5942109

[23] Brennan ME Turner RM Ciatto S Marinovich ML French JR Macaskill P . Ductal carcinoma *in situ* at core-needle biopsy: Meta-analysis of underestimation and predictors of invasive breast cancer. Radiology (2011) 260(1):119–28. doi: 10.1148/radiol.11102368 21493791

[24] Lee JW Han W Ko E Cho J Kim EK Jung SY . Sonographic lesion size of ductal carcinoma *in situ* as a preoperative predictor for the presence of an invasive focus. J Surg Oncol (2008) 98(1):15–20. doi: 10.1002/jso.21077 18459155

[25] Pilewskie M Stempel M Rosenfeld H Eaton A Van Zee KJ Morrow M . Do LORIS trial eligibility criteria identify a ductal carcinoma *in situ* patient population at low risk of upgrade to invasive carcinoma? Ann Surg Oncol (2016) 23(11):3487–93. doi: 10.1245/s10434-016-5268-2 PMC5070657 27172775

[26] Shi B Grimm LJ Mazurowski M Marks J King L Maley C . Can upstaging of ductal carcinoma *in situ* be predicted at biopsy by histologic and mammographic features? Acad Radiol (2017) 24(9):1139–47. doi: 10.1117/12.2255847 PMC5557686 28506510

[27] Bae JS Chang JM Lee SH Shin SU Moon WK . Prediction of invasive breast cancer using shear-wave elastography in patients with biopsy-confirmed ductal carcinoma *in situ* . Eur radiology. (2017) 27(1):7–15. doi: 10.1007/s00330-016-4359-6 27085697

[28] Mann RM Cho N Moy L . Breast MRI: State of the art. Radiology (2019) 292(3):520–36. doi: 10.1148/radiol.2019182947 31361209

[29] Yoon GY Choi WJ Cha JH Shin HJ Chae EY Kim HH . The role of MRI and clinicopathologic features in predicting the invasive component of biopsy-confirmed ductal carcinoma *in situ* . BMC Med Imaging (2020) 20(1):1–11. doi: 10.1186/s12880-020-00494-z PMC7424652 32787871

[30] Heo S Park AY Jung HK Ko KH Kim Y Koh J . The usefulness of ultrafast MRI evaluation for predicting histologic upgrade of ductal carcinoma *in situ* . Eur J Radiol (2021) 136:109519. doi: 10.1016/j.ejrad.2020.109519 33429208

[31] Lee C-W Wu H-K Lai H-W Wu W-P Chen S-T Chen D-R . Preoperative clinicopathologic factors and breast magnetic resonance imaging features can predict ductal carcinoma *in situ* with invasive components. Eur J Radiol (2016) 85(4):780–9. doi: 10.1016/j.ejrad.2015.12.027 26971424

[32] Tanaka K Masuda N Hayashi N Sagara Y Hara F Kadoya T . Clinicopathological predictors of postoperative upstaging to invasive ductal carcinoma (IDC) in patients preoperatively diagnosed with ductal carcinoma *in situ* (DCIS): A multi-institutional retrospective cohort study. Breast Cancer (2021) 28(4):896–903. doi: 10.1007/s12282-021-01225-0 33599914 PMC8213581

[33] Lee SA Lee Y Ryu HS Jang MJ Moon WK Moon HG . Diffusion-weighted breast MRI in prediction of upstaging in women with biopsy-proven ductal carcinoma *in situ* . Radiol (2022) 213174. doi: 10.1148/radiol.213174 36154288

[34] Zhu Z Harowicz M Zhang J Saha A Grimm LJ Hwang ES . Deep learning analysis of breast MRIs for prediction of occult invasive disease in ductal carcinoma *in situ* . Comput Biol Med (2019) 115:103498. doi: 10.1016/j.compbiomed.2019.103498 31698241

[35] Mori N Abe H Mugikura S Miyashita M Mori Y Oguma Y . Discriminating low-grade ductal carcinoma *in situ* (DCIS) from non-low-grade DCIS or DCIS upgraded to invasive carcinoma: effective texture features on ultrafast dynamic contrast-enhanced magnetic resonance imaging. Breast Cancer (2021) 28(5):1141–53. doi: 10.1007/s12282-021-01257-6 33900583

[36] Li J Song Y Xu S Wang J Huang H Ma W . Predicting underestimation of ductal carcinoma *in situ*: a comparison between radiomics and conventional approaches. Int J Comput Assist Radiol Surg (2019) 14(4):709–21. doi: 10.1007/s11548-018-1900-x 30569330

[37] Cohen IJ Blasberg R . Impact of the tumor microenvironment on tumor-infiltrating lymphocytes: Focus on breast cancer. Breast Cancer (2017) 11, 1178223417731565. doi: 10.1177/1178223417731565 28979132 PMC5617083

