# Supplementary material for: Prediction of the histologic upgrade of ductal carcinoma in situ using a combined radiomics and machine learning approach based on breast dynamic contrast-enhanced magnetic resonance imaging
Source: Front Oncol. 2022 Nov 2;12:1032809. doi: 10.3389/fonc.2022.1032809 (PMC9667063; doi:10.3389/fonc.2022.1032809)
Supplement: Supplementary file 1 [file Table_1.docx]

Supplementary Table. List of the extracted features

| Feature Category | | Feature Name |
| --- | --- | --- |
| Shape (14) | | Elongation, Flatness, LeastAxisLength, MajorAxisLength, Maximum2DDiameterColumn, Maximum2DDiameterRow, Maximum2DDiameterSlice, Maximum3DDiameter, MeshVolume, MinorAxisLength, Sphericity, SurfaceArea, SurfaceVolumeRatio, VoxelVolume |
| Intensity (18) | | 10Percentile, 90Percentile, Energy, Entropy, InterquartileRange, Kurtosis, Maximum, MeanAbsoluteDeviation, Mean, Median, Minimum, Range, RobustMeanAbsoluteDeviation, RootMeanSquared, Skewness, TotalEnergy, Uniformity, Variance |
| Texture | GLCM (24) | Autocorrelation, ClusterProminence, ClusterShade, ClusterTendency, Contrast, Correlation, DifferenceAverage, DifferenceEntropy, DifferenceVariance, Id, Idm, Idmn, Idn, Imc1, Imc2, InverseVariance, JointAverage, JointEnergy, JointEntropy, MCC, MaximumProbability, SumAverage, SumEntropy, SumSquares |
|  | GLDM (14) | DependenceEntropy, DependenceNonUniformity, SmallDependenceLowGrayLevelEmphasis, SmallDependenceHighGrayLevelEmphasis, DependenceVariance, GrayLevelNonUniformity, GrayLevelVariance, HighGrayLevelEmphasis, LargeDependenceHighGrayLevelEmphasis, LargeDependenceLowGrayLevelEmphasis, LargeDependenceEmphasis, LowGrayLevelEmphasis, DependenceNonUniformityNormalized, SmallDependenceEmphasis |
|  | GLRLM (16) | GrayLevelNonUniformity, GrayLevelNonUniformityNormalized, GrayLevelVariance, HighGrayLevelRunEmphasis, LongRunEmphasis, RunVariance, LongRunHighGrayLevelEmphasis, LongRunLowGrayLevelEmphasis, LowGrayLevelRunEmphasis, RunPercentage, RunEntropy, RunLengthNonUniformity, ShortRunEmphasis, ShortRunLowGrayLevelEmphasis RunLengthNonUniformityNormalized, ShortRunHighGrayLevelEmphasis, |
|  | GLSZM (16) | GrayLevelNonUniformity, GrayLevelNonUniformityNormalized, GrayLevelVariance, HighGrayLevelZoneEmphasis, LargeAreaEmphasis, LargeAreaHighGrayLevelEmphasis, LargeAreaLowGrayLevelEmphasis, LowGrayLevelZoneEmphasis, SizeZoneNonUniformity, SizeZoneNonUniformityNormalized, SmallAreaEmphasis, SmallAreaHighGrayLevelEmphasis, SmallAreaLowGrayLevelEmphasis, ZoneEntropy, ZonePercentage, ZoneVariance |
|  | NGTDM (5) | Busyness, Coarseness, Complexity, Contrast, Strength |

GLCM= gray-level co-occurrence matrix, GLDM= gray-level dependence matrix, GLRLM= gray-level run length matrix, GLSZM= gray-level size zone matrix, MCC= maximum correlation coefficient, NGTDM= neighborhood gray-tone difference matrix
